# Smoked or Bewitched? The Relationship Between Cannabis Use and Mental Illness Among the Shona Persons in Zimbabwe

**DOI:** 10.1007/s11013-025-09898-4

**Published:** 2025-03-05

**Authors:** Maja Jakarasi

**Affiliations:** Department of Anthropology, University of the Western Cape, Robert Sobukwe Road, Private Bag X17, Bellville, 7535 South Africa

**Keywords:** Mental illness, Cannabis use, Smoked, Bewitched, Zimbabwe

## Abstract

The metanarrative of biomedicine and “psy” discipline (psychology, psychoanalysis, psychiatry etc.) asserts that cannabis use is one of the fundamental causes of mental illness among different men in the Rushinga district of Zimbabwe. These metanarratives, however, appear to have universalised, medicalised and marginalised the conception and representation of mental illness as enmeshed in local epistemologies and ontologies of mental illness. Based on local epistemologies, elders in Diwa largely trace mental illness to discursive sociocultural explanations rarely linked to cannabis use. This paper answers the central question: How is the use of cannabis by different persons related to mental illness in the Rushinga district? I argue that community members, health providers and police officers want to think of persons, especially men, with mental illness as “mad” and immoral cannabis users who brought illnesses upon themselves and lack personal responsibility based on Western neoliberal and biomedical metanarratives. However, this framing is not helpful, it is detrimental to treatment and social reputation, as it bypasses local cultural explanations that may be protective and that offer clearer guidelines for treatment.

## Introduction: Munya Panicking

Munya came dangerously close to where his mother was hand-hoe-weeding and confronted her, "Is today not a *chisi* day!?” His mother was shocked at her son’s behaviour. Munya was then still only fifteen years old and did not fully understand the symbolic meanings of *chisi*. *Chisi* is a day ritually dedicated to spirit mediums (*svikiro*) and ancestral spirits (*midzimu*) at the end of every lunar month. For theologian Shoko (2007, p. 1), it is a shorthand for *chisingarimwi* (no cultivation) among the Karanga Shona-speaking people.

According to anthropologist Bourdillon (1987, p. 256), *chisi* is a sacred time when Shona people are ritually not allowed to weed or cultivate in their fields. Whilst it is a day of staying at home, it is also when ancestral spirits temporarily withdraw their protection to engage in other higher spiritual assignments. Their temporal abandonment leaves “vulnerable people” without spiritual protection from various illnesses (*matenda*), especially people with “mild” mental illnesses like “*mhengera mumba”* (“madness inside the house”)—which often intensifies during “thin moon/month” *(mwedzi mutete*) a time that also coincides with *chisi*. In this context, staying at home during *chisi* is intended to respect ancestral spirits and reduce various spiritual incidents (*madzanzi*) like illnesses whilst enhancing social reproduction.

Munya fled from the field and his mother pursued him. As she was following him, she met his *bamunini* (uncle) who joined her in pursuing her son. When they found him, Munya was hauling obscene and shameful language and trying to climb a very thorny tree. He had incurred several cuts on his body as a result and was bleeding. His mother and *bamunini* could not understand what had possessed or bewitched him. Munya’s *bamunini* suspected that Munya was under the influence of marijuana/cannabis (*mbanje*), and he asked Munya’s mother to find him a razor blade so he could cut incisions (*nyora*) on Munya’s body. It is believed that if someone gets intoxicated from smoking *mbanje* and abruptly sees his blood, this may help in detoxicating him. Munya’s mother could not remember if the *nyora* were cut as she was still confused by the spectacular unfolding of the entire event.

Munya’s friend attested that Munya had never smoked *mbanje*. Munya’s mother is surprised, however, that his condition often deteriorates towards and during the summer season. The mother suspects that if a family member is bewitching Munya, he/she is sacrificing him for an “occultic” farming ritual (*tsvera*) during the farming seasons to ensure a good harvest.

Munya’s mental illness experiences started in 2007 in the Diwa area—the traditional name (*zita revhu/pasi*) of the place under Makuni Chiefdom. Munya is still experiencing mental health issues, which he has chosen to refer to as panicking* (kuvhunduka*). His family, especially his mother, has explored all available healing pathways: traditional medicine, faith healing and biomedicine but none has successfully cured his mental health condition. Munya’s polygamous father works in one of the major towns in Zimbabwe. His polygamous relations had far-reaching implications in the endeavours to heal Munya. According to Shona cultural norms, the father should decide on the health-seeking trajectories of their children. Despite having other children, Munya’s mother seems to have largely refocused on Munya’s condition. She is the only one left determined and committed to helping her son. Some wives in this polygamous family blame the use of cannabis for Munya’s “mental illness”, thus framing the problem as an individual responsibility that must not disadvantage the flow of support within the family.

Local media reports from “professionals” like teachers, doctors, psychiatrists, psychologists and journalists paint a gloomy picture of the risks associated with the use of cannabis in mental health discourse. For instance, The Herald Zimbabwe, a government-owned paper regularly presents stories on the adverse consequences of using *mbanje* on mental illness, as captured in some of these headlines: “excessive use of cannabis” (Nhunzvi, 2019) and “don’t underrate *mbanje* effects” (Chirisa, 2019). Beyond these headlines, cannabis use and mental illness exist as a subject of public concern which regularly features as quick facts and figures across social media as well as in schools, clinics, churches, televisions and radios. Recently, on 19 September 2024, The Herald Zimbabwe revised its previous constructions on the harmful effects of cannabis use, probably because *mbanje* has been legalised in Zimbabwe for medicinal use. The headline reads, “Medical cannabis now on the market”, it describes how the medicine has been adopted by biomedicine to assist with “stress, sleep stress and relaxation”. According to an article in this newspaper, cannabis is expected to give Zimbabwe an annual profit of 20 million USD (The Herald, 2024). It is interesting to note the bias the government has shown in cannabis use based on biomedicine rather than local explanations.

Munya is now routinely using psychiatric drugs to ease his “mental illness”, even though he and his mother believe that his *kuvhunduka* (panicking) is a manifestation of either witchcraft, sorcery, aggrieved spirits (*ngozi*) or spiritual possession that requires prophetic or traditional healing. Whilst Munya’s mother is still uncertain as to whether his mental illness can be attributed to cannabis, many of his close kin and affines, community members and health providers attribute his “situation” to the use of cannabis. This article therefore seeks to explore the central question: How is the use of cannabis by different persons related to mental illness in the Rushinga district? My focus in this article is to explore the discursive relationship between cannabis use and mental illness. I will elaborate on this relationship by unpacking how an individual may explain himself and be seen in mental health. Secondly, I will discuss how a “mad” person is governed by biomedical, and finally, how the local elders, individuals and close family members construct “mental illness”. Based on an anthropological analysis, I argue that biomedical explanations that blame mental health problems on cannabis use are insufficient if attention is not paid to the local meanings communities ascribe to mental health.

### Cannabis Use and Mental Illness: Public Health Perspectives

The World Health Organisation (WHO) defined mental health as a “state of well-being in which an individual realises his or her abilities, can cope with the normal stresses of life, can work productively and can contribute to his or her community” (see Bruun, 2023). DSM-IV, therefore, defines “mental disorders as a clinically significant behavioural or psychological syndrome or pattern that occurs in an individual and that is (e.g. pain symptom) or with a significantly increased risk of suffering death, pain, disability, or an important loss of freedom” (Stein et al., 2010, p. 2). This conception of mental health and mental disorder lies within psychiatry and psychology and overemphasises pathology. Anthropologist Bruun (2023, p. 2) exposed that modern psychology has defined “mental illness” as “interiority subjective” which resides in the “mind” or “brain” of an individual. However, he further argues that mental health is a “question of varied social significance as we move historically and ethnographically across time and space”. Thus, it is historically constructed around notions of pathology and the differentiation between normal and non-normal conduct.

The United Nations Office on Drug and Crime World Drug Report (2023) estimated that globally in 2021 over 296 million people have used substances which is an increase of twenty-three per cent from the previous decade. The number of people who suffer from substance use disorders has skyrocketed to 39.5 million, which is a forty-five per cent increase after a decade. The United Nations Office on Drugs and Crime (2015) estimated that globally 181.8 million people aged between 15 and 64 years used cannabis^1^ for nonmedical purposes (World Health Organization, 2016). This makes cannabis the most used substance by the global population with the highest effects of “cannabis-use disorders^2^” in the world. UNODC argues that the post-legalisation of cannabis in the world has adversely impacted women and youth (UNODC World Report, 2022). Substance use in Africa is on the rise, with projections estimating a forty per cent increase in people who use them between 2018 and 2030. This represents the largest increase globally, with Sub-Saharan Africa projected to have the highest increase when compared to other regions, whilst West Africa is the main supplier of drugs in Sub-Saharan Africa (Marandure et al., 2023). In Africa, youth^3^ represent the largest number of people being treated for drug-related disorders (UNODC World Drug Report, 2022). According to local police officers and health providers, *mbanje* is the most used “drug” by male youths in the Rushinga district.

According to Charles (2023), globally cannabis has been used for centuries and has been criminalised for the past century. In 1955, during colonisation, the use of cannabis was prohibited in Zimbabwe (then Southern Rhodesia) under the Dangerous Drugs Act (DDA) [chapter 15:02] (Rusenga et al., 2024). In 2018, the ban on the use^4^ of cannabis was lifted for industrial and medical purposes only. However, all other uses including recreational smoking are still illegal (Rusenga et al. *Ibid*). Marandure et al. (2023) highlighted that Zimbabwe has gone through decades of significant political and socio-economic challenges which also caused dramatic increases in substance and “drug” abuse. Although there are no official statistics on “drug” and substance consumption in Zimbabwe, some youth organisations paint a gloomy picture (see UNICEF, 2023). Cannabis use is higher in male youths and young adults. Chivandire and January (2016) argued that cannabis use disorders present a growing public health concern and exacerbate existing mental health problems as well as increase injuries, respiratory problems and social problems, such as low educational attainment, criminality, poor interpersonal relationships and risky sexual behaviours in Zimbabwe.

At the same time, the biomedical and “psy” disciplines (psychology, psychiatry, psychoanalysis) posit cannabis use as a major culprit causing skyrocketing mental illness cases across the globe (World Health Organisation, 2016). Amidst the ongoing debate on the use of cannabis and its effects on mental illness, many scholars including Chivandire and January (2016) raised the alarm on the increasing demand for treatment of mental illnesses associated with the use of cannabis in both high- and middle-income countries. Halah et al. (2016) described cannabis as the most used “drug” with major effects for those people with underlying mental illnesses. Atakan (2009) supported that almost half of the people with severe mental illness have used cannabis at some point in their lifetime. There is a growing body of evidence indicating the co-occurrence of depression and cannabis use (see e.g. Feingold & Weinstein, 2021) and depression causing an excessive need to use cannabis. Under these chronic mental conditions, the use of cannabis causes serious consequences for existing mental illness (Halah et al., 2016).

Lowe et al. (2019) have been very sceptical about the continued legalisation of cannabis in Western countries, especially its potential impact on vulnerable persons with mental illness. Dolphin and Newhart (2022, p. 2) warned that the clinical evidence for using cannabis in the treatment of mental illness is limited. The use of cannabis remains formally classified as having no use in medical treatment at the federal level in the USA, whilst its recreational uses are stigmatized (*Ibid*). In a nutshell, the intersection of cannabis and mental health has been highlighted as a controversial relationship. Psychiatry pushed the narrative that cannabis triggers insanity and psychosis (*ibid*). Budney et al. (2019) summed these complexities and the multidimensional relationship between cannabis and mental illness in this ongoing global debate as confusion and controversies regarding whether cannabis use causes harm or provides benefits.

### Anthropological Perspectives on Mental Health

Meanwhile, anthropologists have extolled a holistic approach of ethnographic perspectives that helps traverse and reconcile psychiatry and anthropology by inserting ethnographic insights within and beyond clinical settings (e.g. Bruun, 2023; Kleinman, 1988). Anthropologists Kohrt & Mendenhall (2016) shared that culture is fundamental in mental health discourse, as it influences symptoms, causes, and treatment of mental illness which are unique and distinct from other health problems. Mental health therefore squarely resides in the methods, theories and questions of anthropology. This Anthropologist Janzen and his colleague Arkinstall, a medical doctor also affirmed the importance of lay therapy management groups which are usually composed of families, relatives, kids or friends in their “quest of therapy” (Janzen & Arkinstall, 1978). Medical anthropology allows us to take a holistic conception of the body (individual, social and body politic) to unpack the concealed experiences and meanings in “illness narratives” (see e.g. Scheper-Hughes & Lock, 1987; Kleinman, 1988) articulated by natural metaphors (Kirmayer, 1992). Therefore, based on an anthropological analysis, Munya’s story may help in the “emic determination of disorder” (Marsella 1978, p. 351) and “detailed local phenomenological descriptions” (Kleinman et al., 1978, p. 4) of relationships between “mental illness” and cannabis use. That is the individual and local cultural explanations of different mental health experiences are fundamental, particularly in exposing the relationship between mental illness and cannabis use.

## Methods

I conducted ethnographic fieldwork from February 2024 to April 2024**.** My fieldwork was in the Nyamanyanya area, under the Diwa area, in the Rushinga district, Mashonaland Central province. Diwa is a traditional name for the area under the jurisdiction of Chief Makuni (see Jakarasi, 2013, pp. 19–20), adjacent to the southeastern Tsombe chiefdom where I grew up. This is an area where my mother was born. Therefore, I have long frequented this area during holidays to see my matrilineal grandfather (*asekuru* Achopuchopu), grandmother (a*mbuya* Anyanda) and other relatives of the Shawa *mainje* clan name. I chose this study area to make a follow-up on Munya’s mental condition which I witnessed when I was still teaching at one of the schools in that area. During my visits to Diwa, I used to see Munya often as we had become friends. We used to spend our time together whilst he furnished me with all the local developments. Munya had a sharp memory, and he became a key informant.

I also interviewed eight participants: a traditional healer, a “mentally ill” person and his mother, a traditional leader—Chief Makuni, three adolescents, a nurse and a psychiatrist. I purposively selected two traditional healers (based on their perceived abilities to heal mental illnesses), one mentally ill person based on his mental health experiences and condition and the mother, based on her relationship and lastly the three adolescents, a female and two males, also chosen based on their experiences in *mbanje* use. I used semi-structured interviews to collect qualitative data.

I was granted ethical clearance by the Humanities and Social Science Research Ethics Committee of the University of the Western Cape. The committee approved the methodology and ethics of this study under HS23/9/39. I was further granted permission to conduct fieldwork in the Diwa area by Chief Makuni under whose jurisdiction my participants reside. All information was kept confidential, by anonymising the names of the participants except for Chief Makuni who chose his name to be published. As sensitive as the issue of mental illness is, I was careful when I was talking with my participants.

### Biomedical Explanations of the Relationship Between Mental Illness and Cannabis Use

Based on my fieldwork, the local self-explanatory models (see Kleinman et al., 1978) of those individuals experiencing “mental illnesses” and those of close family members are fundamental in narratives of mental health. It highlights unique meanings and experiences based on a local shared understanding of illnesses. The findings and discussions below will interrogate the entanglements between cannabis use and “mental illness” experiences in the contemporary Rushinga rural district of Zimbabwe. I will first explore how Munya, and his mother understand his experiences.

#### Individual Self-Explanations of Cannabis Use and Mental Illness

Based on pervasive and dominant neoliberal and biomedical narratives, several people in the Diwa community perceive and understand Munya’s experiences as psychiatric disorders, attributed to the use of cannabis. General perceptions by the community are based on pervasive biomedical metanarratives popularized by media, education institutions and Christianity in the 21st century. However, Munya understands his experiences as direct manifestations of witchcraft and spirits, where his close kin or affine are engaged in sorcery for illicit wealth accumulation, which ultimately inflicts him into panic. Although his mother’s understanding of his situation was skewed to local explanations, she also hardly rules out the use of *mbanje*. She is always quoted, “Munya must also stop smoking so that we can also be certain on where his illness is coming from”.

Anthropologists note that individuals accept or resist, for instance, name-callings that underlie “sinful” perceptions about mental illness (see Bruun, 2023), such as those commonly deployed by insensitive community members. Although many traditional healers, psychiatrists and nurses in the Diwa community often use “*kupenga”* (“madness”) or *“chirwere chepfungwa”* (“mental illness”) as a shared cultural understanding that explains mental health experiences, Munya resisted these concepts and accepts ‘*kuvhunduka’* (panicking), whilst his mother generalised his health condition just as *kurwara* (illness). Many people especially those who see him at the local shopping centre, where he often relaxes and accesses *mbanje* address him with different unfortunate delegatory names like *vhundu* (slang for panicking), *mujambanje* (notorious eater of *mbanje)*, or *tsviru or mupengo* (dangerous “lunatic”). These names unseparated, denigrate and marginalise his ‘*kuvhunduka*’ condition. They rationalised his “panics” as adverse effects of smoking *mbanje*. Therefore, the community rejects Munya as reckless with his life, ill-mannered, evil, or dangerous to the children. However, the most intricate characteristic of the story was that some of Munya’s colleagues who supplied *mbanje* or smoked with him were hardly misconstrued as persons with “mental illness”. Unlike some of these unsensitive community members or his colleagues, Munya however appreciated our friendship because I always addressed him by his real name, Munya, and his situation as ‘*kuvhunduka*’, a concept he preferred and perceived to better explain his condition.

Whilst *‘kuvhunduka*’ was not a universal or common local concept used to describe mental illness experiences in Diwa, it was a concept that Munya used to describe his illness. Munya carefully chose this term to eschew the precarity associated with mental illness and *mujambanje.* Munya suffered stress, self-rejection and self-unworthiness from some community members. For Munya, the notion of *kuvhunduka* explains how he “panics” whenever he is being attacked by witchcraft familiars. Despite his acceptance of *kuvhunduka,* this notion has never been a perfect experience for Munya. It was a creation of witchcraft that was harming his life. Contemporary, very few people in Diwa wanted to be addressed as *kuvhunduka* because of how the concept has been used to explain Munya’s experiences which resonated with labellings levelled against him based on his health challenges. *Kuvhunduka* has acquired new negative meanings associated with “madness” and the reckless smoking of *mbanje*.

As Munya’s condition continued to deteriorate, he preferred to be understood as simply *munhu akadhakwa* (drunk person) rather than *munhu anovhunduka* (panicking person), because some community members have transformed *kuvhunduka* into the slang form *vhundu* (the act of panicking) to describe him as “madman”. It can be noted that the notions and concepts preferred by those people experiencing “mental illness” are transitory and require a great level of sensitivity.

The factors that pushed Munya into smoking can be encapsulated in his account: “Now I just want people to say I am drunk, not mad. Being seen as mad is embarrassing and shameful. They used to say I am intoxicated by *mbanje*, at least if I smoke, they will just say I am drunk. I have seen that those who are intoxicated are viewed better than me. I can also forget about relatives bewitching me and gather confidence to fight them”. The use of *mbanje* has never made his discrimination and stereotypes better. He acquired a new name, *mujambanje* (reckless consumer of *mbanje*), which makes Munya the sole architect of his mental illness. Munya’s ‘*kuvhunduka*’ further highlights how complicated relationships, names and notions in cannabis use and mental illness can be created. For Kleinman (1988), this is what he has described as rhetorical skills of individuals in deploying idioms sometimes to escape shame associated with their symptoms. However, these skills have always met some resistance from the community.

In the prolegomenon of this article, Munya’s mother and *bamunini* construct Munya’s experiences, as “psychosis” attributed to excessive smoking of *mbanje* by someone too young. Although cutting of incisions (*nyora*) on the body as a way of trying to detoxicate individuals from *mbanje* is not a shared and generally accepted cultural understanding, it gives us meanings on how community especially close kins or affines can easily construct “mental illness” as the manifestation of cannabis use. In contrast, Munya articulated his side of the story which refuted the association of his condition with cannabis use*.* He recalls that his *kuvhunduka* had started before he even started smoking *mbanje*. Munya explained

My situation started well before many people [including his parents] knew it. The people who first noticed it were my classmates. I went to school, and my colleagues, said I was speaking too much, not that I was speaking louder but speaking without listening or giving others time to speak. Christine, one of my classmates was the first to notice it. She went to tell her mother that the way I was talking too much (*kutaurisa*) was problematic. I was panicking (*kuvhunduka*) and my heart was beating faster. I am not mentally ill (*kupenga*) but its *kuvhunduka*. I had not started smoking *mbanje* when I started *kuvhunduka*. My situation did not start in the field that my mother always referenced. Yes, she first saw it when I was in the field on *chisi* and suspected that it was *mbanje*. I started smoking later after five years from this field episode.

Based on this account, Munya started smoking *mbanje* to heal himself from existential precarity: “depression”, stress, “anxieties”, stereotypes and discrimination which arose from some insensitive community members. He had already started speaking louder and panicking.

#### Governing the “Mad”: Smoked or Bewitched?

Generally, several community members, health providers and police officers attribute various mental illnesses of young men to excessive use of substances. Despite a shared local and cultural understanding of mental illness, many community members seem to be shifting their explanations of mental illness towards cannabis use—governed by powerful biomedical metanarratives. This appears to resonate well with Foucault (1988) who argues that the control, surveillance and treatment of “mad” is often governed by prevailing institutional powers, where biomedicine decides and constructs how “mad” persons are governed.

When Munya was smoking cannabis, he torched his mother’s thatched kitchen and missed his sister with an axe. Expectedly, he was arrested for an act of arson and attempted murder. The police officers who arrested him attributed this incident to smoking *mbanje*, whilst on the other hand, his mother perceives that witches wanted her son to murder her sister, destroy properties and later avail him to perish in prison. In support of her perceptions, before this incident occurred, the mother had dreamed of her daughter swimming in red water, which she symbolically interpreted to foretell the death of her daughter, the sister to Munya. She strongly believed the interpretations of her dreams and how prayers and ancestral spirits had helped dissipate the severity of this hatched witchcraft plan by one of Munya’s close kins.

It can be noted that the construction of the relationship between *mbanje* use, and mental illness is often obscured by legal and pathological narratives. It too deconstructs the local construction of ‘*kuvhunduka*’ experiences and privileges “mental illness” metanarratives. Subsequently, when Munya was attending court for the crime, psychiatrists and psychologists later diagnosed that he had some underlying mental illness—a “relapse” produced by excessive use of *mbanje*. He was transferred to Parerenyatwa Psychiatric Unit, a major hospital in Harare, Zimbabwe. At this point, Munya’s experiences were transformed from legal (smoked) to pathological objects (mental illness), based on biomedicine perspectives. Whilst the diagnosis of mental illness was a positive step for the treatment of mental illness, the phenomenological understanding of Munya and the local explanation of ‘*kuvhunduka*’ was missing in the whole gamut of explanations and treatments. *Kuvhunduka* that resonated with witchcraft.

Munya explained his experiences at psychiatric hospitals as corresponding to that of prison or police detention he had initially endured in the hands of police officers for “his crime”. The *Kuvhunduka* narrative from Munya during his “confinement” has never been tolerated and has been misconstrued as “madness”. Except for some psychiatrists and psychologists, few people wanted to listen to his *kuvhunduka* story. The interpretation of Munya’s health by local nurses in Diwa and psychologists and psychiatrists was related to smoking *mbanje*, thus making his “condition” a wilful intent. Munya explains

People lied that I was in a psychiatric hospital. That one was not a hospital. We all agreed with some of my colleagues that it was just a prison for smokers. We were all the times accused of smoking *mbanje*. We were always taught how to stop smoking when we were “set free’. They called it ‘restoration’. You know *kusina amai hakuyendwi* (Shona proverb: you should not go where there is no mother). I also stopped smoking because I had no access to *mbanje*. But the panicking has never stopped, although some officers said I was getting better.

Munya’s mother was happy with the condition of Munya soon after he was released from the psychiatric hospital where he had stayed for almost a year. Although Munya wanted not to stay away from his mother who understood his condition, the mother was somehow impressed with how her son has improved his “hygienic”. As soon as Munya was released from the psychiatric hospital, his mother attempted to agree with the rest of the community that the condition of his son was attributed to smoking *mbanje*. However, this was not the case, the condition of Munya was still alive. This short-lived acceptance of the health improvement of Munya by his mother can be best explained by anthropologist Bourgois (1995) who argues that the social suffering alienates and naturalises substance use and ultimately pushes people to view illness as their architect. Munya’s mother explained

When he “relapsed”, the psychiatrist told him he had been smoking *mbanje*. Now, I don’t know maybe he was far away from his witches or it's this *mbanje*. Witches could go everywhere, or he was not being used in farming for *tsvera*. I stay with my son; many people are even saying it's smoking. I am praying that he stops so that those witches hiding behind substance abuse can be exposed.

Possibly, the temporal “normal responses” from the psychiatric unit by Munya were achieved after he stopped smoking *mbanje* or was based on the treatment received. Despite these biomedical narratives, the re-emergence of “*kuvhunduka*” may resemble *mhengera mumba* (“madness” inside the house) a Shona mental health construction. According to Gelfand (1967, p. 42), *mhengera mumba* is a type of mental illness where a person gets “mentally ill” when he/she is in his/her village but recovers when that person is away from witches’ influence. The illness also reemerges when he/she returns to their influence. Thus, witchcraft and spirits become important places for also elaborating the relationship between cannabis use and mental illness for Munya.

### Witchcraft and Spirits

Despite the dominant biomedical explanations offered on mental illness experiences, witchcraft and spirit symbols are important metaphors and phenomenological descriptions for Munya, his mother and some community members. Following many interviews with Munya and some interlocutors, new insights were opened on thin moon worldviews, aggrieving spirits, sorcery, witchcraft and spiritual calling which complicates the discursive relationships between mental illness and cannabis use. Although these sociocultural constructions rarely altogether lead to mental illness, they rather show different social bodies where mental illness could come from. These nuances highlight the subjectivities and complexities situated beyond smoking cannabis. If the story by Munya which reveals that he had started *kuvhunduka* before smoking *mbanje* holds, it therefore becomes pertinent to explore beyond the implicit assumptions widespread in the biomedical framing that overemphasises the nexus of cannabis use and mental illness.

#### Mwedzi Mutete and Mental Illness

The citing of *chisi* in the introduction may be interpreted as incantations curated by spirits of *chisi*. To understand cultural nuances associated with *chisi*, I interviewed Chief Makuni, the autochthonous of the land (*varidzi vepasi*), concerning mental illness. His conception below further unsettles cannabis use.

When the month is about to end or start, this period is referred to as *nhinda*, *huwakwedza* or *chisi*. This occurs when the moon becomes thin (*mwedzi mutete*). It is the time when people are prohibited from working in their fields by spirit mediums, and several women go to the menstrual cycle (*kuyenda kumwedzi*-literally translated to the moon). They are cleansed to be fertile. However, *mwedzi mutete* is associated with several “extremes” for people especially those with chronic mental illnesses. This is the time when many of them become violent or too silent. The spirit mediums would have stopped providing their close spiritual protection as they would be attending to other spiritual commitments. This is the time when different illnesses wreak havoc. People must rest to avoid encounters with misfortunes and afflictions.

Chief Makuni reiterated the sacredness of cultivating fields during *chisi*. Anthropologist Bourdillon (1987) pointed out that this time is authoritatively designated by Chiefs and enforced by *mhondoro*. For Muchinako et al. (2013), *mwedzi mutete* (thin moon) is the time that coincide with *chisi*, and is associated with *mhengera mumba* (mild mental illness). Persons with “unstable and mild mental illness” becomes “violent”. These persons are affected for a few days and get better as the moon gets bigger. The severity of *mhengera mumba* is marginal. For some elders, it implies that the “madness” is still marginal (close inside the house (*mumba*)). However, for Gelfand (1967, p. 42), it implies that a person gets “mentally ill” when they are close to the village where the influences of witches are strong.

Munya’s *kuvhunduka* experiences often “deteriorate” during *chisi*. It appears, that Munya’s underlying ‘*kuvhunduka*’ responds to the effects of “*mwedzi mutete*”. The behaviour of Munya in the field appears to have been coupled with disobeying the taboo of *chisi* and the arrival of *mwedzi mutete*. However, many people disobey this taboo without consequences of mental illness, because they don’t have an underlying mental illness. However, the constructions of mental illness during *mwedzi mutete* situates Munya’s kuvhunduka as a spiritually caused illness.

In a local weekly Shona newspaper, Kwayedza, journalist Mapupu (2019) wrote salient ethnographic insights on *mwedzi mutete*. He found out that when people are saying *mwedzi mutete*, they are signalling deep mental illness meanings. It either means a person is mentally ill, or the mental illness is at its peak because of the embodied “thin moon”. This local ontology of *mwedzi mutete* echoes the observation of psychiatrist Kirmayer (1992) who argues that illness experiences are articulated through metaphors. Scheper-Hughes and Lock (1987, p. 21) supported that “natural phenomenon”, human artefacts, animals and topography are important in classifying and humanising human experiences. It is noted that notions of spirits, experience and time refute the individual responsibility that stresses the individual, particularly demonstrated by smoking *mbanje*, as an artefact of mental illness. In this context, an individual agency of smoking cannabis is deconstructed by the agency of the spirits (see Niehaus, 2002).

#### Avenging Spirits (Ngozi) and Spiritual Possession

*Ngozi*, aggrieved spirit, has since been the mostly feared spiritual phenomenon in Diwa area. It unleashes various sociocultural afflictions and misfortunes, especially by causing fast and cruel deaths, illnesses, miscarriages and divorces among others. Munya’s explanation of his own experiences can be explained as one of the manifestations of *ngozi*:

Many *n’angas* (traditional healers) told me that smoking *mbanje* will never cause permanent mental illness. My brother Tindo was once possessed (*akasvikirwa*) by the spirit of our aunt who died long ago without children. As he was possessed, he told my father that my situation was an act of witchcraft. The person behind my illness had seen that I was bright in school and wanted to disrupt my progress in education. He also said I was in the process of becoming *mudzimu* (spirit medium). When his spirit had gone, elders failed to know whether I was possessed, mentally ill, bewitched or smoking. Some also said that *ngozi* from our late aunt who died without children, because of sorcery within the family was coming to haunt us. This *ngozi* spirit has never been appeased. Elders believe a *ngozi* within the family, is less life-threatening. The one from another family is too harmful. This kind of *ngozi* may cause symptoms like my “panicking” experiences, for family members to search for meanings and appease.

My brother from my stepfather also got mentally ill. He started when he was in the shop. He indiscriminately and violently looted groceries from the shop. We were all victims of witchcraft pervasive in our extended family. It was family issues. We have many family challenges: witchcraft *(huroyi*) and sorcery (*kuromba*). Contrary to my situation, my brother has been healed by the prophet Dollar and is now doing well. When I also visited Dollar, he said I was too late (*ndakanonoka*), as my situation had deteriorated, and I encountered Western medicine. My Father was told by Chayambuka, one of the renowned healers, who once tried to heal me in Tsombe that *mbanje* will never cause *kupenga* “madness”. He also said my situation is never related to smoking *mbanje*. Still, smoking *mbanje* was later used by witches or sorcerers in my family to avoid accounting for my situation.

Munya’s account also highlights spiritual possession and *ngozi* discourses that situate mental illness in traditional Shona cosmology. In Shona cosmology, once “*ngozi*” emerges, it must be paid for restorative justice between individuals, families, and the community. However, failure to appease “*ngozi*” results in inexplicable misfortunes such as illnesses or sudden deaths (Musanga, 2017, p. 775).

Mental illness related to the process of becoming a traditional healer has never been spared. Some elders in Diwa suspected that Munya might have been in the process of becoming a traditional healer hinted by a possessed sibling. Mental illness is often one of the characteristics of the liminal or marginal phase of spiritual transition undergone by traditional healers in their ‘rites of passage’ (See Turner, 1982). This liminal phase is locally referred to as *kusuka homwe* (cleansing medium). Mental illness experiences during spiritual transformative processes are not new among anthropologists, they have constructed these mental illnesses that can only end when one accepts the spiritual calling (Bourdillon, 1987, p. 164; Luedke, 2007, p. 716; Reis, 2000, p. 62; Janzen, 1992, pp. 139–140; Turner, 1982, p. 282).

Munya, including his mother’s accounts, highlights witchcraft, which frequently features in family affairs. Explanations by Munya’s brother linked his illness to witchcraft, particularly invoked by jealousy of Munya’s perceived intelligence at school and being one of the most loved children in a polygamous family. This had raised witchcraft accusations at the family level.

Some of Munya’s relatives were perceived to be engaging in “occult” farming magic (*tsvera/divisi*) to enhance their yields. The mother of Munya constructs this kind of mental illness as a ritual for accumulating illicit wealth. As sufferers “panic” and wander at the shopping centre, sorcerers will also accumulate more wealth. Furthermore, Munya’s mother also notices some profound increases in the severity of mental illness of her son, especially during the summer season when peasant farmers start engaging in farming preparations. Munya stays at the shopping centre but frequently relocates to the homestead whenever the rain season begins. This norm points to a systematic illness being controlled by “someone living” (*munhu mupenyu*)*—*a “sorcerer”. Munya would have been called by goblins to engage and invoke farming charms.

My interview with Pachawo, one of the *n’angas* in Diwa, about mental illness experiences and cannabis use, revealed an inextricable proliferation of sorcery which has gained traction in recent decades. She explains

These young people are engaged in sorcery (*kuromba*) of *zvikwambo* and *zvidhoma* for accumulating wealth, and power, and starting businesses and or even buying posh cars. These charms are detrimental to the health and lives of children. Do you see the level of greediness that has become common in societies? Young people are interested in wealth and money, and this is affecting their children. Witchcraft is very tricky; it can influence you to get infected with HIV/AIDS or even smoke *mbanje*.

Pachawo echoes the detailed phenomenological experiences of Munya related to sorcery. In her narrative, she subtly mentioned smoking *mbanje* as one of the factors which has worsened mental illness cases in Diwa but has immensely pointed to an increased appetite for illicit accumulations.

Sorcery for illicit accumulation is not a new social phenomenon in Zimbabwe. Z*vikwambo* (goblins) or *zvidhoma (*zombies*)* are well-known familiars bought or sold to accumulate wealth through supernatural means but causing illnesses in the family (Shoko, 2007, pp. 64–67), normally mental illnesses. Similarly, in South Africa, Niehaus et al. (2001, pp. 51–56) pointed out that *tokolotsi* and *mamlambo* require blood and human sacrifices from their kin. Anthropologists described this as an “occult economy” (Comaroff & Comaroff, 1999), premised on real or imagined “illicit accumulation” in South Africa. Anthropologists Sanders (2003) and Pfeiffer (2002) cited disruption of social cohesion, that invoked sorcery idioms particularly in Tanzania and Mozambique produced by Structural Adjustment Programmes that heightened competition and inequalities. Mental illness, therefore, becomes an anti-social ritual, through which sorcerers gain political and economic power.

## Conclusion

This article demonstrates discursive relationships between cannabis use and mental illness in the Rushinga district. Based on the “mental illness” experiences of Munya, these relationships could be complex, bidirectional, or mere creations of biomedicine. Munya’s story highlights how rural communities are becoming obsessed with popular biomedical metanarratives from health providers which privilege and pathologize normal experiences. Luckily, close family members like a mother remain understanding, sympathetic and supportive of their close kin in suffering and healing. By their understanding, they are not easily drawn into conjectures. Biomedical metanarratives ostensibly define various mental illnesses as direct manifestations of cannabis use thus making them an individual responsibility. Cannabis use becomes a shorthand for dismissing the existing “mental illness” by shifting the blame to the “sufferer”. For anthropology, the challenge lies in thinking beyond this implicit assumption widespread in biomedicine that conceptualises mental illness as only pathological, individual acts and wilful intent.

Despite the efficacy of traditional healing for mental illness (Jakarasi, 2024), there is a growing missing link from individuals, close relatives and elders concerning diagnoses, symptoms, causes and treatment of mental illness especially if cannabis use is suspected. Whilst cannabis use, based on biomedical framing, is linked to both positive and negative consequences for different persons, a complete disregard for cultural and individual explanations could be detrimental in an endeavour to ensure lasting healing of mental illness. Therefore, biomedical explanations that describe cannabis use and mental health are insufficient, they can obscure and blur cultural realities such as witchcraft and spirits that fundamentally interwove local mental illness experiences. Ignoring local and individual understanding may prolong mental health challenges or present serious consequences to persons with “mental illness”. The individual and cultural explanations of mental illness, for instance, are important in determining causes as well as treatment. Establishing the root cause of mental illness is fundamental in health-seeking behaviours in mental health (Jakarasi, 2024) and may help deal with the root cause rather than addressing its consequences which may be in the form of cannabis use.

## Footnotes

1 “Cannabis is a generic term used to denote the several psychoactive preparations of the cannabis plant (World Health Organization, 2016).

2 “Cannabis-use disorders refer to a spectrum of clinically relevant conditions and are defined via psychological, social and physiological criteria to document adverse consequences, loss of control, overuse and withdrawal symptoms. Cannabis-use disorders are defined in the *Diagnostic and statistical manual of mental disorders* and in the *International statistical classification of diseases and related health problems*” (World Health Organization, 2016).

3 Youth are young people who are between the ages of 15 to 35 (African Youth Charter) (African youth Charter, 2006).

4 Cannabis use mainly involve smoking, and it can also be cooked and drunk and or eaten with some foods.
